# 2-Deoxy-d-Glucose Treatment Decreases Anti-inflammatory M2 Macrophage Polarization in Mice with Tumor and Allergic Airway Inflammation

**DOI:** 10.3389/fimmu.2017.00637

**Published:** 2017-06-01

**Authors:** Qingjie Zhao, Zhulang Chu, Linnan Zhu, Tao Yang, Peng Wang, Fang Liu, Ying Huang, Fang Zhang, Xiaodong Zhang, Wenjun Ding, Yong Zhao

**Affiliations:** ^1^State Key Laboratory of Membrane Biology, Institute of Zoology, Chinese Academy of Sciences, Beijing, China; ^2^Laboratory of Environment and Health, College of Life Sciences, University of Chinese Academy of Sciences, Beijing, China; ^3^College of Life Sciences, University of Chinese Academy of Sciences, Beijing, China; ^4^Department of Urology, Beijing Chaoyang Hospital, Capital Medical University, Beijing, China

**Keywords:** macrophage polarization, M2 macrophages, glycolysis, 2-deoxy-d-glucose, allergic airway inflammation, tumor

## Abstract

As important effector cells in inflammation, macrophages can be functionally polarized into either inflammatory M1 or alternatively activated anti-inflammatory M2 phenotype depending on surroundings. The key roles of glycolysis in M1 macrophage polarization have been well defined. However, the relationship between glycolysis and M2 polarized macrophages is still poorly understood. Here, we report that 2-deoxy-d-glucose (2-DG), an inhibitor of the glycolytic pathway, markedly inhibited the expressions of Arg, Ym-1, Fizz1, and CD206 molecules, the hall-markers for M2 macrophages, during macrophages were stimulated with interleukin 4. The impacted M2 macrophage polarization by 2-DG is not due to cell death but caused by the impaired cellular glycolysis. Molecular mechanism studies indicate that the effect of 2-DG on M2 polarized macrophages relies on AMPK-Hif-1α-dependent pathways. Importantly, 2-DG treatment significantly decreases anti-inflammatory M2 macrophage polarization and prevents disease progression in a series of mouse models with chitin administration, tumor, and allergic airway inflammation. Thus, the identification of the master role of glycolysis in M2 macrophage polarization offers potential molecular targets for M2 macrophages-mediated diseases. 2-DG therapy may have beneficial effects in patients with tumors or allergic airway inflammation by its negative regulation on M2 macrophage polarization.

## Introduction

Macrophages are critical for host immunity and can display different activation states depending on the surrounding contexts (1, 2). Polarized macrophages can be distinguished as M1 and M2 macrophages in response to infections with microorganisms and host mediators (3, 4). Interferon-γ in combination with agonists of Toll-like receptors induces M1 (classical) activation, whereas interleukin 4 (IL-4) or IL-13 promotes M2 (alternative) activation (4, 5). From the host-defense standpoint, M1 macrophages are inflammatory and can serve a positive role in immunity to microbial pathogens and tumors. In contrast, M2 macrophages promote tissue repair and metabolic homeostasis and serve key roles in immunity to parasitic helminthes (5). M1 and M2 macrophages have distinctive metabolic phenotypes that significantly differ from those of resting macrophages (6–8). M1 macrophages greatly rely on aerobic glycolysis, whereas M2 macrophages mainly utilize fatty acid oxidation (FAO; β-oxidation) to fuel mitochondrial oxidative phosphorylation (9). These studies have provided compelling evidence that macrophage polarization can be regulated by the different aspects of cellular metabolism.

2-Deoxy-d-glucose (2-DG) has long been used as an antagonist of glucose metabolism (10). After 2-DG enters into the cells, 2-DG is phosphorylated by the glycolysis rate-limiting enzyme hexokinase (HK) to 2-DG-phosphate (2-DG-P). However, unlike G-6-P in cells, 2-DG-P cannot be subsequently metabolized by phosphohexose isomerase, which converts G-6-P into fructose-6-phosphate (11). The accumulated 2-DG-P leads to glycolysis inhibition predominately at the step of phosphorylation of glucose by HK. The 2-DG-mediated inhibition of the glycolysis rate-limiting step leads to a depletion of cellular ATP so as to block cell cycle progression and cause cell death (12). 2-DG inhibits mitochondrial ATP production and activates AMPK by altering cellular AMP: ATP ratios (13). The inhibition of HK and glycolysis eventually leads to metabolic stress and cellular depletion of ATP (14). 2-DG treatment shifted the cellular rates of oxygen consumption (OCR) and lactate production by extracellular acidification to a predominantly oxidative phosphorylation-dependent metabolism (14). M1-polarized macrophage activation by LPS is dependent on glycolysis. The inhibition of glycolysis by 2-DG significantly decreases the inflammatory response of M1 macrophages (15–17). A critical consequence of succinate accumulation in LPS-activated macrophages is the cell metabolic reprogramming and the induction of IL-1β (18). Blocking glycolysis with 2-DG significantly limits this signal process by decreasing succinate via induction of succinate dehydrogenase (18). IL-1β secretion requires transcription of pro-IL-1β and the process of NLRP3/caspase-1 inflammasome. The functional NLRP3 inflammasome formation requires glycolysis and is significantly inhibited by 2-DG (19). Whether 2-DG treatment impacts anti-inflammatory M2 polarization is unclear. Here, we report the crucial role of glycolysis in anti-inflammatory M2 macrophage polarization by AMPK-Hif-1α-dependent manner and found that 2-DG treatment inhibited M2 macrophage polarization *in vivo* and significantly prevented the development of allergic airway inflammation and tumor growth in mice.

## Materials and Methods

### Animals

C57BL/6 (B6) mice were obtained from Beijing University Experimental Animal Center (Beijing, China). Mice were maintained in a specific pathogen-free facility. All experimental manipulations were undertaken in accordance with the Institutional Guidelines for the Care and Use of Laboratory Animals, Institute of Zoology (Beijing, China) and were approved by the Committee for Animal Care and Use in Institute of Zoology.

### Reagents

Anti-mCD11b-PE-Cy5 and anti-mCD206-PE were purchased from BD Biosciences PharMingen (San Diego, CA, USA). Anti-F4/80-FITC was procured from Tianjin Sungene Biotech (Tianjin, China). Recombinant mouse IL-4 was purchased from PeproTech (Rocky Hill, NJ, USA). The primary antibodies against IRF4 and arginase1 were purchased from Cell Signaling Technology. All of these antibodies were diluted at 1:1,000 in PBS with 5% bovine serum albumin. Anti-β-Actin mAb (1:50,000) was purchased from Sigma-Aldrich.

### Cell Preparation

Bone marrow cells were harvested and cultured with DMEM containing 10% (v/v) FBS and 10 ng/mL of mouse M-CSF for 7 days to obtain bone marrow-derived macrophages (BMDMs) (20, 21). Primary mouse peritoneal macrophages were obtained from the peritoneal exudates of 4–6-week-old mice. The peritoneal exudate cells were washed twice with PBS solution and adjusted to 1 × 10^6^ cells/mL in DMEM cultured for 3–4 h at 37°C and 5% CO_2_ (22). The non-adherent cells were removed by washing with warm PBS. The purification of macrophage was analyzed by flow cytometry (Beckman, CA, USA), using mouse macrophage markers CD11b and F4/80. The adherent cells constituted more than 90% of CD11b^+^F4/80^+^ macrophages.

### Arginase Assay

The arginase (Arg) activity assay was performed as described previously (23, 24). Briefly, the cells were lysed in 0.1% Triton X-100. Tris–HCl was then added to the cell lysates at a final concentration of 12.5 mM, and MnCl_2_ was added to obtain a 1 mM final concentration. The Arg was activated by heating for 10 min at 56°C, and the l-arginine substrate was used at a final concentration of 250 mM. The reactions were incubated at 37°C for 30 min and stopped by the addition of H_2_SO_4_/H_3_PO_4_. After the addition of a-isonitrosopropiophenone and heating for 30 min at 95°C, the urea production was measured as the absorbance at 540 nm, and the data were normalized to the total protein content.

### Cell Death Assay

Cell death was measured using the Annexin V-FITC apoptosis detection kit (Abcam, Mountain View, CA, USA), according to the manufacturer’s instructions. After treatment with IL-4 and/or 2-DG for 48 h, the cells were harvested and washed twice with cold PBS (pH = 7.4). The cells were then incubated with 200 µL binding buffer containing Annexin V-FITC (40 µL/mL) and propidium iodide (PI; 1 µg/mL) for 15 min at room temperature in the dark. The population of PI and Annexin V-positive cells was analyzed by flow cytometry (Epics XL, Beckman Coulter Inc., Pasadena, CA, USA).

### Lactate Dehydrogenase Assay

In order to determine the cellular toxicity of 2-DG, the levels of lactate dehydrogenase (LDH) released from macrophages were measured. After 48 h exposure of IL-4 and/or 2-DG treatment, cell-free supernatant aliquots were separated cells in each experimental sample by centrifuge, and supernatants were transferred to clean flat-bottom plate for enzymatic analysis. LDH in the culture supernatants was measured using commercially available LDH cytotoxicity detection kit-PLUS (Roche Applied Science, Mannheim, Germany). All samples were assayed by a microplate spectrophotometer (Thermo MK3, MA, USA).

### Chitin Administration

Chitin (Sigma) was washed three times in PBS and then sonicated with a UR-20P device (Tomy) for 30 min on ice. After filtration with 100 µM cell strainer, chitin was diluted in 50 mL PBS. About 800 ng chitin with or without 2-DG (1,000 mg/kg) was intraperitoneally injected. The peritoneal macrophages were collected 2 days after administration.

### Quantitative PCR Analysis

Total RNAs were isolated with Trizol (Invitrogen), and reverse transcription was performed with M-MLV superscript reverse transcriptase according to the manufacturer’s instructions. Real-time PCR kit (SYBR Premix Ex Taq™, DRR041A) was purchased from Takara Bio Inc. PCR was done on CFX96 (Bio-Rad). To determine the relative mRNA expressions in response to IL-4, the mRNA expression levels of Arg, Fizz, Ym1, and CD206 were normalized to the housekeeping gene hypoxanthine phosphoribosyl transferase (25). Each sample was determined at least in triplicates. Primers used in the present study for the amplification were summarized in Table 1.

**Table 1:** Primers used in real-time PCR.

Genes	Primer sequence (5′ → 3′)
Argainse1	Forward primer: CCAGAAGAATGGAAGAGTCAGTGT
	Reverse primer: GCAGATATGCAGGGAGTCACC
Ym1	Forward primer: CAAGTTGAAGGCTCAGTGGCTC
	Reverse primer: CAAATCATTGTGTAAAGCTCCTCTC
FIZZ	Forward primer: CTGCCCTGCTGGGATGACT
	Reverse primer: CATCATATCAAAGCTGGGTTCTCC
IRF4	Forward primer: CTTTGAGGAATTGGTCGAGAGG
	Reverse primer: GAGAGCCATAAGGTGCTGTCA
HK II	Forward primer: TTTCACCTTCTCCTTCCCTTGC
	Reverse primer: CACATCTTCACCCTCGCAGC
PKM2	Forward primer: GGTGTTTGCATCTTTCATCCG
	Reverse primer: GAATCTCAATGCCCAGGTCAC
LDH	Forward primer: CATTGCAGTACAGTCCACACT
	Reverse primer: TTCCAATTACTCGGTTTTTGGGA
HPRT	Forward primer: AGTACAGCCCCAAAATGGTTAAG
	Reverse primer: CTTAGGCTTTGTATTTGGCTTTTC

*LDH, lactate dehydrogenase; HPRT, hypoxanthine phosphoribosyl transferase*.

### Western Blot Assay

The peritoneal macrophages were cultured in DMEM medium with 10% FCS in 12-well plates. Cells were treated with IL-4 and the indicated reagents for the indicated time depending on the experiment purpose. After stimulation, the cells were washed once in cold PBS, lysed in RIPA buffer (50 mM Tris–HCl pH 7.4, 1% NP-40, 0.25% Na-deoxycholate, 150 mM NaCl, 1 mM EDTA pH7.4) with protease and phosphatase inhibitor cocktails (Sigma) for 10 min on a rocker at 4°C. Cells were scraped, centrifuged at 12,000 rpm for 10 min at 4°C, and the supernatants were mixed with 5× protein-loading buffer (26). Protein concentration is determined using a BCA assay. Proteins samples were analyzed on SDS-PAGE and transferred onto polyvinylidene difluoride (PVDF) membrane (Millipore). PVDF membrane was blocked with TBST (100 mM Tris–HCl pH7.5, 150 mM NaCl, 0.05% Tween20) with 5% non-fat dried milk for 1 h and then incubated with primary antibodies overnight on a shaker at 4°C. The appropriate HRP-coupled secondary antibody was used and detected through chemiluminescence (Millipore) (27). β-Actin was used as a protein-loading control. PVDF membranes were stripped, washed in TBST, and immunostained with the other antibody.

### Glycolysis Flux Assay

Glycolysis of macrophages was detected by measuring the detritiation of [3-^3^H]-glucose. In brief, the assay was initiated by adding 1μCi [3-^3^H]-glucose (Perkin Elmer) for 2 h. Medium was transferred to microcentrifuge tubes containing 50 µL 5 N HCL. The microcentrifuge tubes were then put in 20 mL scintillation vials containing 0.5 mL water and the vials capped and sealed. ^3^H_2_O was separated from un-metabolized [3-^3^H]-glucose by evaporation diffusion for 24 h at room temperature.

### Measurements of Glucose and Lactate

A total of 2 × 10^6^ cells per well were seeded in 12-well plates for 2 days. The medium was collected, and the glucose and lactate levels were examined immediately. Glucose and lactate were measured spectrophotometrically using an Olympus AU5400. The glucose consumption and lactate production were normalized to cell numbers.

### Tumor Model

B16-F1 melanoma (B16) cells (1 × 10^6^, in 200 µL PBS) were injected subcutaneously into the flanks of mice. 2-DG treatment was once per day from 9 days (1,000 mg/kg) (28). Male C57BL/6 strains of mice (6–8 weeks) were used. There was no systematic means of randomization of mice, and the experiment was carried out blindly throughout. The mice were sacrificed on day 19. Tumors were resected and transferred to 5 mL PBS on ice. Tumor weight was measured on a scale. The tumors were then processed for flow cytometry sorting on the same day or fixed in formalin for immunohistochemistry.

### OVA-Induced Allergic Airway Inflammation

OVA-induced acute allergic inflammation was elicited as previously described by i.p. sensitization with 20 mg OVA mixed with 2 mg alum at days 1 and 14 (22, 29). From day 21, mice followed by nebulizer-delivered airway challenges with 1% OVA for 5 consecutive days. The mice were pretreated 2 days before aerosolized and intraperitoneally injected 2-DG (1,000 mg/mL) continuous till the sacrifice. Mice were assessed 24 h after the last OVA challenge. Cells from bronchoalveolar lavage fluid (BALF) were stained with anti-mSiglecF, anti-mCD11c, anti-mCD206, anti-mCD11b, and anti-mCD45 mAbs and analyzed by a flow cytometry. The expressions of M2 markers including Arg, Fizz, Ym1, and CD206 in these cells were detected by real-time PCR. Lung tissues were fixed with formaldehyde and embedded in paraffin. The thin sections of the embedded tissues were stained with hematoxylin and eosin (H&E) stain. The evaluation of inflammatory infiltrate was done microscopically.

### Bronchoalveolar Lavage Preparation

Bronchoalveolar lavage fluid was performed according to the method of Haque et al. (30). In brief, the lungs were lavaged *in situ* three times with 1.5 mL cold PBS. Recovered BALF was immediately cooled to 4°C and centrifuged at 1,700 rpm for 5 min (27). The BALF cells were stained for CD45^+^CD11c^+^SiglecF^+^ and sorted by a MoFlo XDP Cell Sorter (Beckman). The purification of alveolar macrophages was up to 98%.

### Statistical Analysis

All data are presented as the mean ± SD. Statistical significance was determined by two-tailed Student’s *t*-test or by one-way ANOVA. Data were analyzed using one-way ANOVA followed by *post hoc* comparisons using the Tukey’s multiple or two-way ANOVA statistical analysis followed by a *post hoc* test. A *p* value less than 0.05 was considered as statistically significant.

## Results

### M2 Macrophage Polarization Depends on Glycolysis

**Figure 1:**
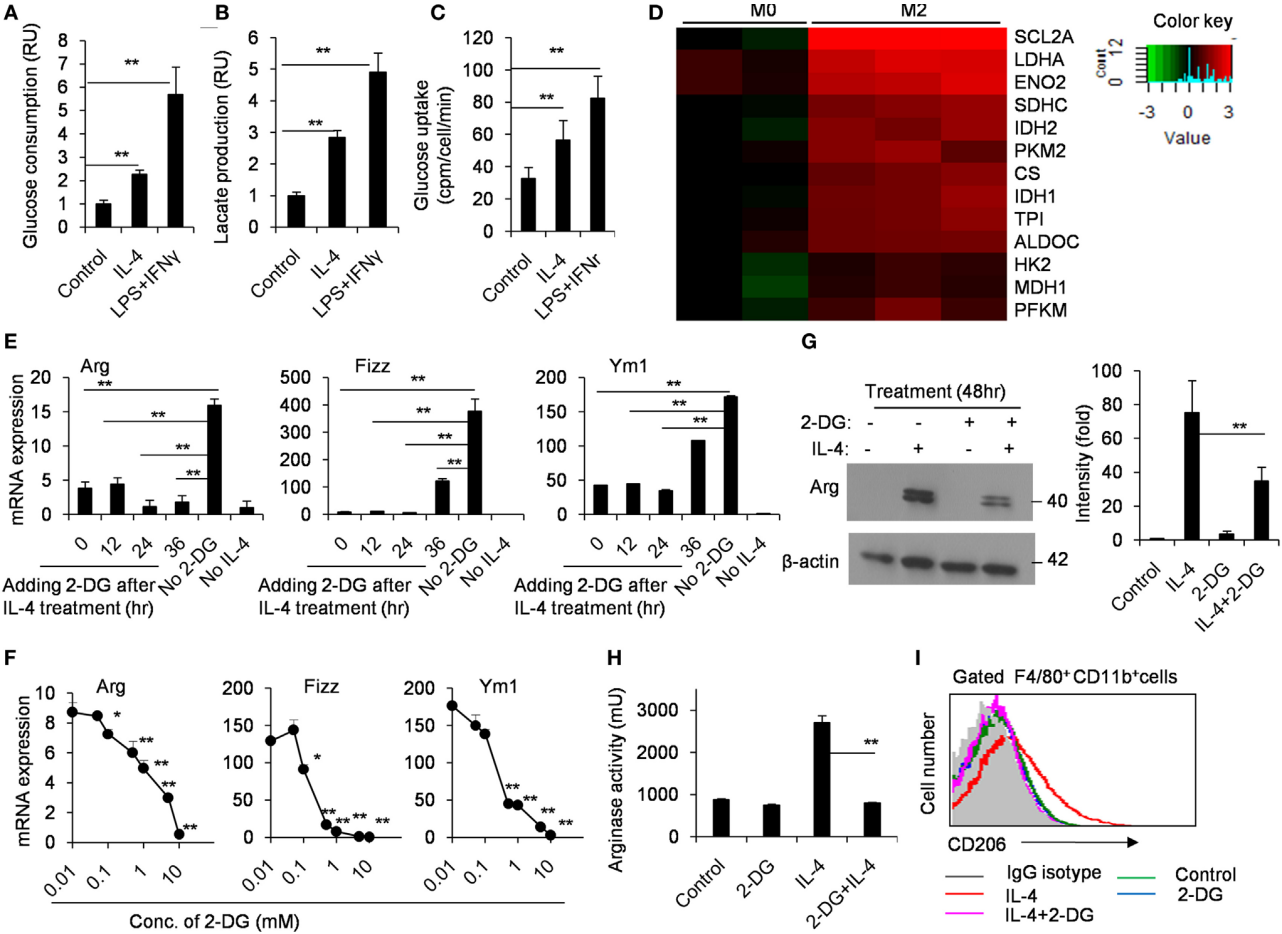
M2 macrophages depend on glycolysis. Culture media of control and interleukin 4 (IL-4)-stimulated peritoneal macrophages were collected for the measurement of glucose **(A)** and lactate **(B)**. **(C)** The freshly isolated peritoneal macrophages were treated with IL-4 (1,000 U/mL) for 48 h. The glycolytic activity of these cells was measured by the generation of ^3^H-labeled H_2_O from [3-^3^H]-glucose. **(D)** The glycolysis-related molecules were determined by real-time PCR after peritoneal macrophages were stimulated with IL-4 for 48 h. **(E)** Gene expressions of Arg, Fizz, and Ym1 in peritoneal macrophages activated with IL-4 for 48 h and 2-deoxy-d-glucose (2-DG) treatment added at 0, 12, 24, and 36 h after cell culture were measured by quantitative real-time PCR. **(F)** Macrophages were treated with 0.01, 0.1, 1, and 10 mM 2-DG for 1 h and stimulated with IL-4 for 48 h. The mRNA expression of Arg, Fizz, and Ym1 was determined by real-time PCR. **(G)** Peritoneal macrophages were exposed to 1 mM 2-DG for 1 h and stimulated with IL-4 for 48 h. Cell lysates were blotted for arginase. **(H)** Peritoneal macrophages were treated with 2-DG for 1 h and stimulated with IL-4 for 48 h. The activity of arginase was determined by fluorescence. **(I)** Flow cytometry analysis of CD206 expression on IL-4-induced M2 macrophages with the exposure of 2-DG. Experiments were done more than two times. Data were shown as mean ± SD (*N* = 4). *p < 0.05, **p < 0.01 compared with the indicated group or the control.

It has been well appreciated that M1-polarized macrophages increased glycolysis to quickly trigger microbicidal activity. However, how glycolytic activity is regulated in M2 macrophage polarization was poorly understood. Thus, we compared the glucose consumption and lactate production in M0 and M2 macrophages using freshly isolated peritoneal macrophages from naïve B6 mice. As shown in Figure 1, M2 macrophages had an enhanced glycolysis (*p* < 0.01, Figures 1A,B). The glycolytic uptake of M2 macrophages was measured by the generation of ^3^H-labeled glucose from [3-^3^H]-2-DG. Our results showed that M2 macrophages contained much higher glycolytic uptake than M0 macrophages (*p* < 0.01, Figure 1C). Glucose utilization depends on a chain of reactions catalyzed by multiple key enzymes, eventually leading to the generation of lactate and net production of two ATP molecules as the energy source. Real-time PCR analysis revealed that IL-4-stimulated M2 macrophages markedly increased these genes encoding glycolysis-related molecules, including the transporter Glut1 (Slc2a1), hexokinase II (HK II) and aldolases (Aldoc) (*p* < 0.01, Figure 1D). These data collectively indicate strong upregulation of glucose metabolism during IL-4-induced M2 macrophage polarization. To directly test the importance of the metabolic reprogramming in M2 macrophage activation, we induced M2 polarization in the presence or absence of 2-DG. While IL-4 stimulation for 48 h, macrophages were treated with appropriate doses of 2-DG, and their mRNA expression profiles were analyzed over a time course ranging from 12 to 36 h using real-time PCR. We found that the mRNA expression of Arg, Fizz, and Ym1 reduced even 2-DG treatment was employed as later as 36 h after IL-4 induction (*p* < 0.01, Figure 1E). Similarly, the IL-4-induced mRNA expression of Arg, Fizz, and Ym1 was substantially diminished as the concentrations of 2-DG increasing, indicating that 2-DG inhibits M2 polarization in a dose-dependent manner (*p* < 0.01, Figure 1F). Additionally, 2-DG treatment resulted in decreased production and activity of Arg by IL-4-induced M2 macrophages (*p* < 0.01, Figures 1F,G). Furthermore, 2-DG severely reduced the expression of CD206, a cell surface marker for M2 macrophages, on IL-4-treated macrophages as determined by flow cytometry assays (*p* < 0.01, Figure 1I; Figure S1 in Supplementary Material). Together, these results suggest that glycolysis is necessary and significant for macrophage polarization and low doses of 2-DG treatment blocks M2 macrophage polarization in a time- and dose-dependent manner.

### Effects of 2-DG on Glycolysis in M2 Macrophages

**Figure 2:**
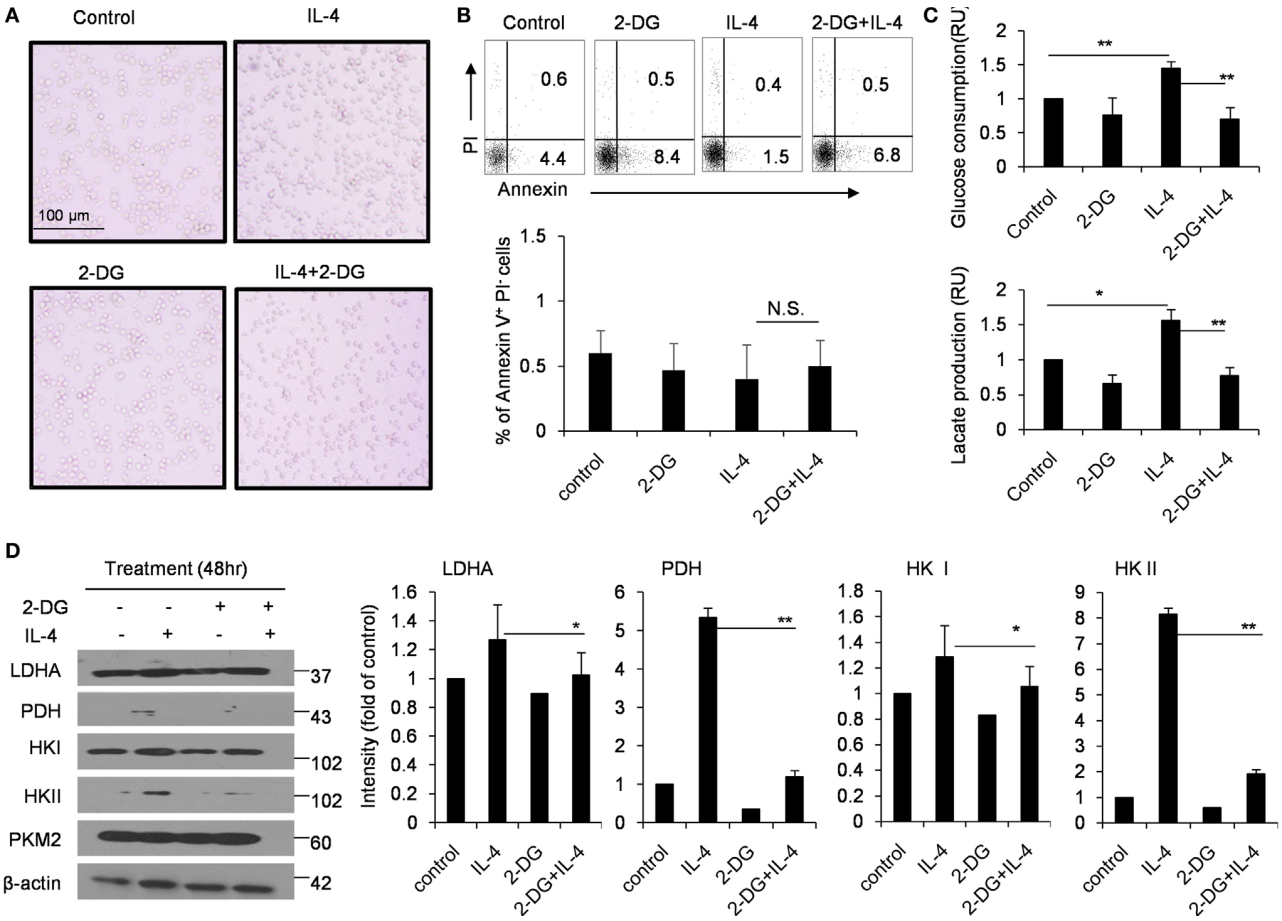
Effects of 2-deoxy-d-glucose (2-DG) on cell death and glycolysis in M2 macrophages. **(A)** Morphological images of peritoneal macrophages exposed to interleukin 4 (IL-4) with or without 1 mM 2-DG for 48 h. **(B)** Flow cytometry results of the Annexin V-FITC and propidium iodide (PI) assays. The peritoneal macrophages were treated with 1 mM 2-DG for 1 h with or without IL-4 for 48 h. **(C)** Culture media of IL-4-stimulated bone marrow-derived macrophages with or without 2-DG treatment were collected for the measurement of glucose and lactate. **(D)** The levels of LDHA, PDH, HK I, HK II, PKM2, and β-actin in peritoneal macrophages after 2-DG (1 mM) and IL-4 (1,000 U/mL) treatment for 48 h were detected by Western blots. The dot plots and the relative mean values have been obtained from a single representative experiment of experiments that gave very similar results. Data are shown as mean ± SD (*N* = 4). *p < 0.05 and **p < 0.01 compared with the indicated group.

In order to exclude the possibility that the decreased M2 polarization caused by 2-DG is simply caused by the increased cell death, we detected the cell death ratio after macrophages were treated with as high as 1 mM 2-DG *in vitro*. We observed 2-DG treatment did not remarkably alter the cell activity of M2 macrophages (Figure 2A). Furthermore, M2 macrophages treated with or without 1 mM 2-DG were harvested and stained with Annexin V and PI. Flow cytometry less than 7% of 2-DG-treated M2 macrophages showed Annexin V^+^PI^−^, and approximately 1% of the cells were Annexin V^+^PI^+^, which is similar to 2-DG-untreated macrophages (Figure 2B). Moreover, the level of LDH released from 2-DG-treated macrophages was similar with M0 and IL-4-treated macrophages (Figure S2 in Supplementary Material). These results excludes the possibility that cell death contributes to the poor M2 macrophage polarization caused by the treatment with low doses of 2-DG.

The glucose consumption and lactate production were also significantly inhibited by 2-DG treatment during IL-4-induced M2 polarization using the *in vitro* M-CSF-induced BMDMs, as observed in freshly isolated peritoneal macrophages (*p* < 0.01, Figure 2C). The cellular requirements for energy can be supplied by the enzymatically regulated glycolysis of glucose into glucose-6-phosphate, accompanied by the release of ATP; an endpoint of this process is the release of pyruvate from cells which is the enzyme substrate for TCA cycle. Glucose process depends on a chain of reactions catalyzed by multiple important enzymes. Thus, we used Western blots to detect the expressions of HK II and PDH in M2 macrophages after 2-DG treatment. Consistent with the decreased glucose consumption, 2-DG treatment significantly decreased the HK II and PDH protein expressions in M2 macrophages (*p* < 0.01, Figure 2D). These results showed that 2-DG treatment could significantly impact the glucose metabolism states of M2 macrophages.

### Glycolysis Controls M2 Polarization in an AMPK-Hif-1α-Dependent Fashion

**Figure 3:**
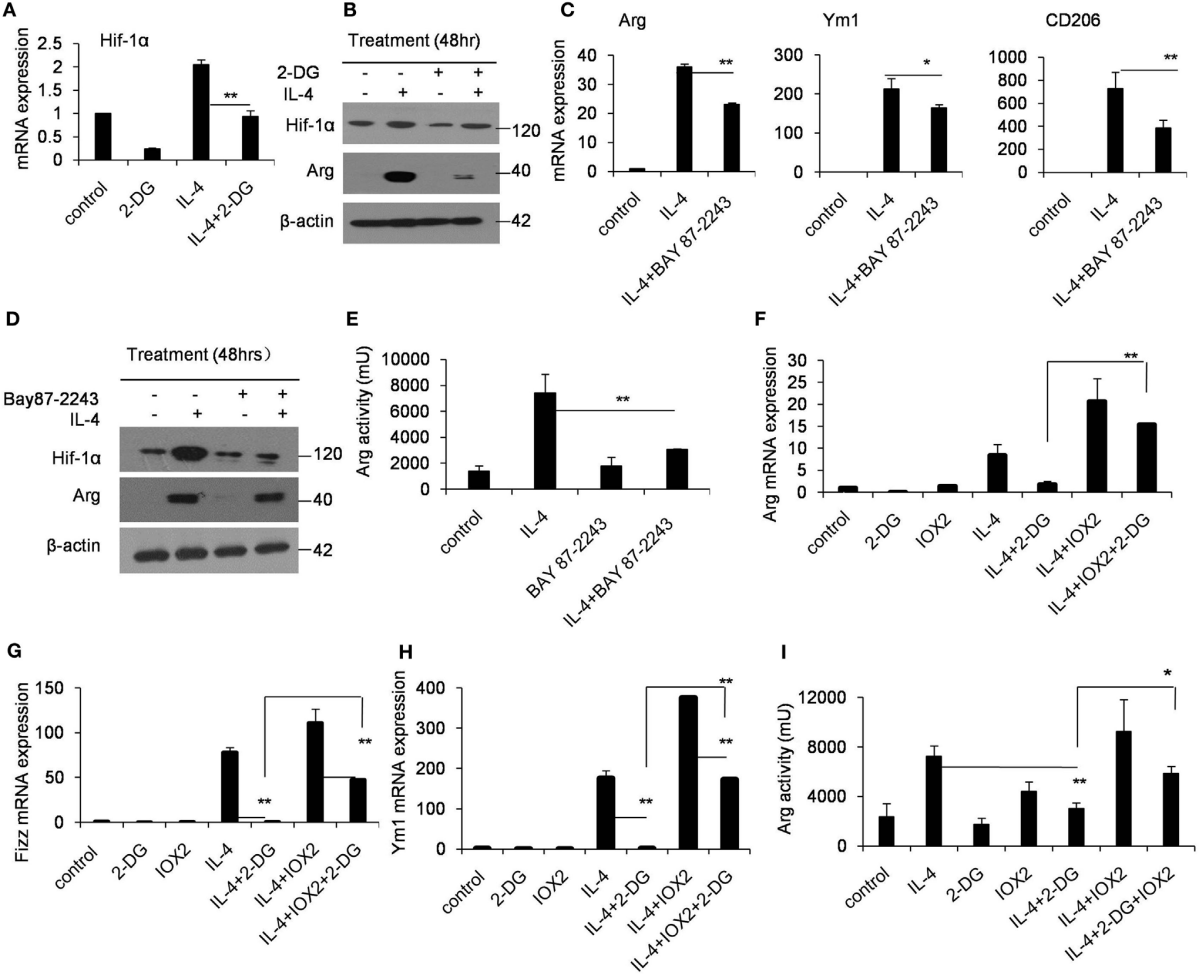
Glycolysis controls M2 polarization in a Hif1α-dependent fashion. **(A)** The expression of Hif-1α in peritoneal macrophages was determined by real-time PCR after pretreated with 2-deoxy-d-glucose (2-DG) for 1 h and stimulated with interleukin 4 (IL-4) for 48 h. **(B)** Macrophages were pretreated with 2-DG for 1 h and stimulated with IL-4 for 48 h. Hif-1α expression was determined by Western blots. **(C)** The peritoneal macrophages were treated with or without Bay 87-2243 (10 µM) for 1 h and then treated with IL-4 stimulation for 48 h, respectively. The mRNA expression levels of Arg, Ym1, and CD206 were determined by real-time PCR. **(D)** The macrophages were treated with Bay 8702243 (10 µM) *in vitro* for 1 h and stimulated with IL-4 for 48 h. The Hif-1α, arginase, and β-actin protein levels were determined by Western blots. **(E)** The activity of arginase was determined by fluorescence after macrophages were treated with Bay 8702243 and IL-4. The mRNA expression of Arg **(F)**, Fizz **(G)**, and Ym1 **(H)** was determined by real-time PCR. The macrophages were treated with IOX2 (10 µM) for 1 h and stimulated with 2-DG and IL-4 for 48 h. **(I)** The activity of arginase was determined by fluorescence after macrophages were pretreated with IOX2 (10 µM) and then stimulated with 2-DG and IL-4 for 48 h. Experiments were done more than two times. Data were shown as mean ± SD (*N* = 4). **p < 0.01 compared with the indicated group.

**Figure 4:**
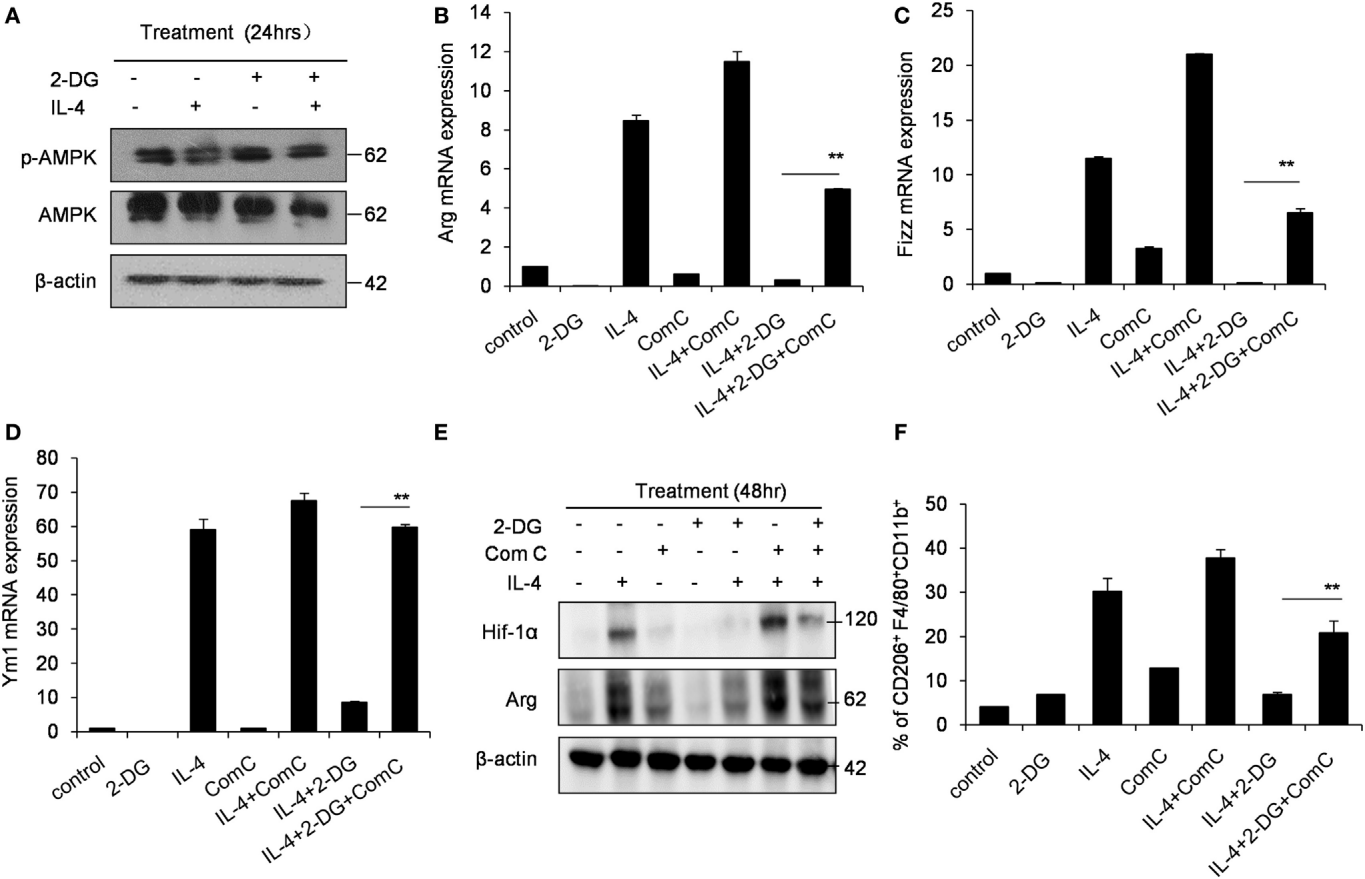
Glycolysis controls M2 polarization in an AMPK-Hif1α-dependent pathway. **(A)** The activity of p-AMPK in peritoneal macrophages were detected by Western blotting after 2-deoxy-d-glucose (2-DG, 1 mM) and interleukin 4 (IL-4, 1,000 U/mL) treatment for 24 h. Inhibition of AMPK by compound C (1 µM) significantly enhanced the expressions of Arg **(B)**, Fizz **(C)**, and Ym1 **(D)** in macrophages stimulated with IL-4 for 48 h as determined by real-time PCR. **(E)** The level of Hif-1α, arginase and β-actin in IL-4-stimulated peritoneal macrophages with or without compound C (1 µM) was determined by Western blots. **(F)** The decreased CD206 + F4/80 + CD11b + cells by 2-DG was partially rescued by compound C (1 µM) during IL-4 stimulation, which were determined by flow cytometry. Experiments were done more than two times. Data were shown as mean ± SD (*N* = 4). **p < 0.01 compared with the indicated group.

Hif-1α is a transcription factor crucial for the induction of a M2-like macrophage polarization and controls the expression of genes encoding rate-limiting components of the glycolytic pathway (31). Additionally, the functions of Hif-1α are most well defined in relation to modifying glycolysis (32, 33). To assess whether Hif-1α regulation is involved in the poor M2 macrophage polarization caused by 2-DG, we therefore detected the Hif-1α expression in macrophages treated with 2-DG. The expression of Hif-1α was increased in IL-4-induced M2 macrophages. However, 2-DG treatment significantly inhibited the IL-4-induced Hif-1α mRNA expression in macrophages as detected by real-time PCR (*p* < 0.01, Figure 3A; Figure S3 in Supplementary Material). Nicely consistent with the mRNA expression, we detected less expression of Hif-1α in macrophages treated with IL-4 and 2-DG than those in macrophages treated with IL-4 alone by Western blots (Figure 3B). It is reported that Bay 87-2243 is a Hif-1α inhibitor (34). Bay 87-2243 treatment significantly decreased the expressions of M2 makers like Arg, Ym1, and CD206 in IL-4-induced M2 macrophages as assayed by real-time PCR (*p* < 0.01, Figure 3C). The Arg protein expression and activity were also decreased after Bay 87-2243 treatment as detected by Western blots (Figures 3D,E). In addition, as the treatment of Hif-1α activator, IOX2 (35, 36), for 48 h could significantly reverse the 2-DG-impaired expressions of M2 makers including Arg, Ym1, and CD206 molecules as determined by real-time PCR (*p* < 0.01, Figures 3F–H). Similarly, IOX2 treatment rescued the decreased activity of Arg in M2 macrophages under 2-DG treatment (*p* < 0.01, Figure 3I). As a suppressor of tumorigenesis and inflammation, AMPK activation opposes most of the metabolic alterations that occur in proliferating cells (37). Furthermore, it has been shown that 2-DG administration had effects on AMPK activation (38). Thus, we detected the activation of AMPK during M2 macrophage induction in the presence and absence of 2-DG. The phosphorylation of AMPK in macrophages was decreased after IL-4 treatment. However, 2-DG treatment rescued this alteration as determined by Western blots (Figure 4A; Figure S4 in Supplementary Material). It is reported that compound C inhibits AMPK activation (39, 40). Compound C treatment significantly rescued the 2-DG treatment-decreased expressions of Arg, Fizz, and Ym1 molecules during M2 macrophage induction as detected by quantitative PCR (*p* < 0.01, Figures 4B–D). In consistent with mRNA levels, we found that the inhibition of AMPK under compound C treatment could upregulate expression of Hif-1α and arginase in the presence of 2-DG as determined by Western blots (Figure 4E). 2-DG treatment significantly decreased the percentage of CD206^+^ cells in F4/80^+^CD11b^+^ macrophages in the presence of IL-4 (*p* < 0.01, Figure 4F). However, compound C treatment could significantly rescue the decreased percentage of CD206^+^ cells in F4/80^+^CD11b^+^ macrophages in the presence of IL-4 in the presence of 2-DG as determined by flow cytometry (*p* < 0.01, Figure 4F). Therefore, the deficiency of AMPK activation induced Hif-1α expression and then enhanced M2 macrophages polarization.

### Crucial Role of Glycolysis in M2 Macrophage Polarization Caused by Chitin

**Figure 5:**
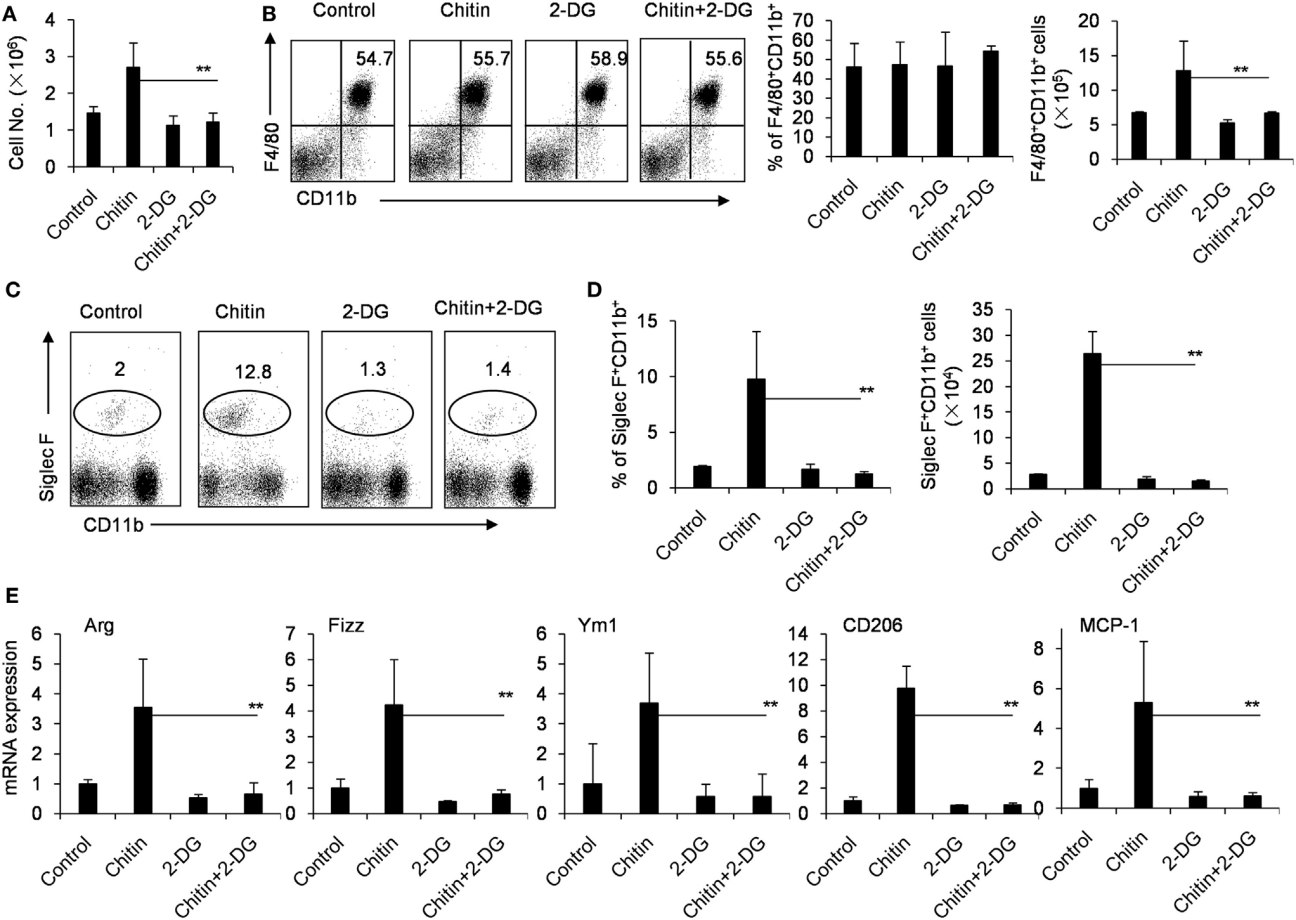
Crucial role of glycolysis in M2 macrophage polarization in response to chitin administration *in vivo*. **(A)** The cells of peritoneal cavity were harvested from mice 2 days after peritoneal injection of chitin with or without 2-deoxy-d-glucose (2-DG, 1,000 mg/kg). The total cell numbers were counted. The expressions of CD11b, F4/80, or Siglec-F on the cells of peritoneal cavity were determined by flow cytometry assay. The percentages and numbers of F4/80^+^CD11b^+^ macrophages **(B)** or Siglec-F^+^ CD11b^+^CCR3^+^ eosinophils **(C,D)** in the cells of peritoneal cavity harvested from mice 2 day after peritoneal injection of chitin with or without 2-DG (1,000 mg/kg) were shown. **(E)** Quantitative PCR showing expression of Arg, Ym1, Fizz, CD206, and MCP-1 mRNAs in the freshly isolated peritoneal macrophages. Total RNA of peritoneal macrophages was prepared 48 h after mice received administration of chitin with or without 2-DG. Eight mice in each group were assayed. Data were shown as mean ± SD (*N* = 8). **p < 0.01 compared with indicated group.

Chitin, a polymerized sugar, is a structural component of helminths, arthropods and fungi (41). Chitin administration recruits M2 macrophages to the administration location, which are very important for the following recruitment of eosinophils (42, 43). Indeed, the intraperitoneal administration of chitin significantly recruited more cells, including F4/80^+^CD11b^+^ macrophages and Siglec-F^+^CD11b^+^ eosinophils to the peritoneal cavity after 48 h (Figures 5A–D). However, 2-DG treatment decreased the total cell number of peritoneal cells, and importantly, the cell numbers of the recruited Siglec-F^+^CD11b^+^ eosinophils after chitin treatment (Figures 5A,C,D). Consistent with this observation, Chitin-elicited peritoneal macrophages expressed higher levels of Arg, Fizz and Ym1, the hall-markers of M2 macrophages (44), than control peritoneal macrophages isolated from chitin-untreated mice (*p* < 0.01, Figure 5E). However, 2-DG treatment considerably reduced the expression of genes encoding Arg, Fizz, Ym1, CD206, and MCP-1 in chitin-induced peritoneal macrophages compared with those of only chitin-elicited mice (*p* < 0.01, Figure 5E). These results suggest that 2-DG prevents M2 macrophage polarization in response to chitin administration *in vivo*.

### 2-DG Reduces M2 Macrophages Polarization in Tumor-Bearing Mice

**Figure 6:**
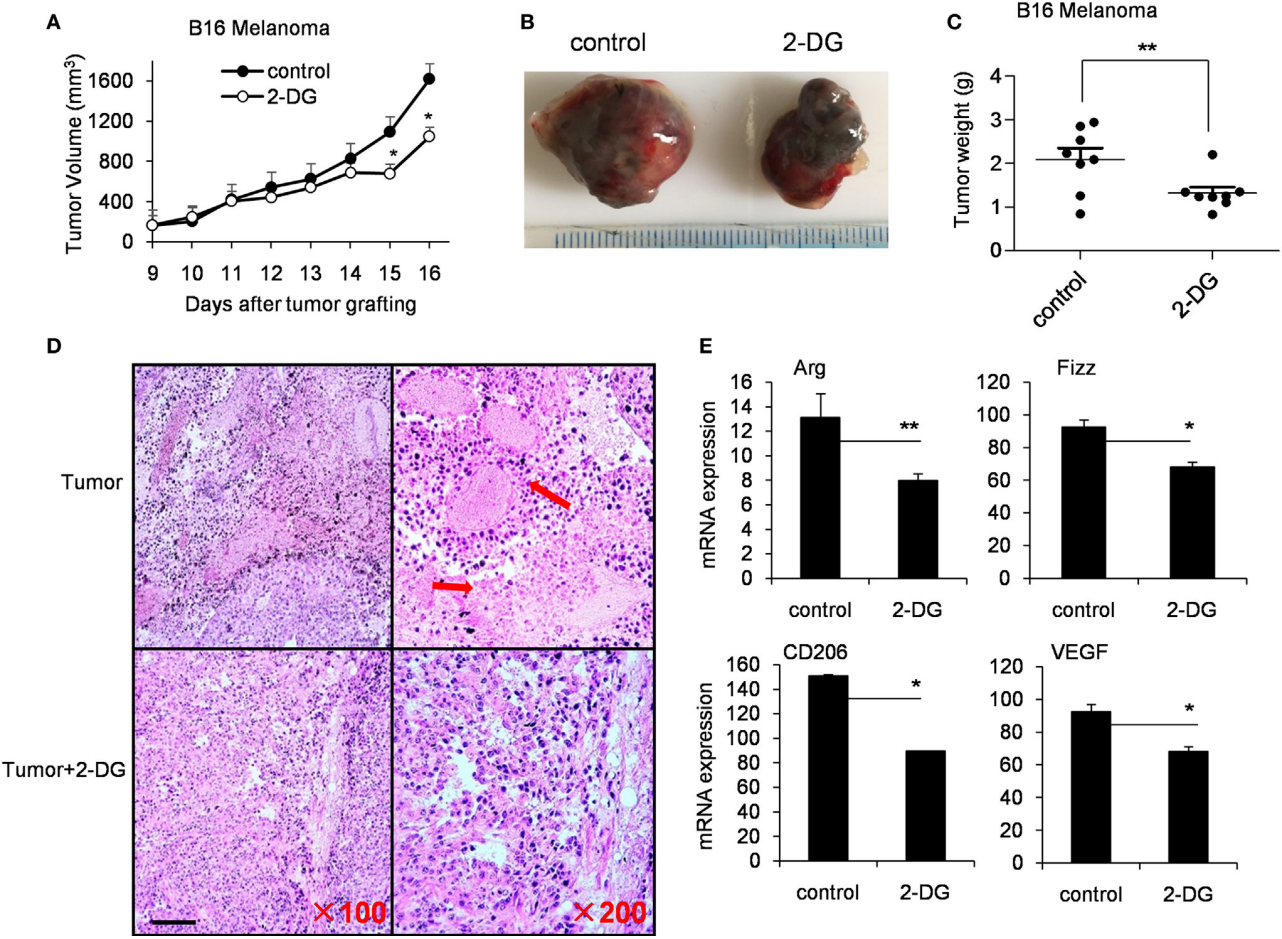
2-Deoxy-d-glucose (2-DG) treatment inhibited tumor growth and reduced M2 macrophage polarization. **(A)** Tumor growth curve in mice with injections of 2-DG. **(B)** Representative images of tumors harvested from mice in each treatment cohort. **(C)** Average tumor weights on day 14 after the first 2-DG treatment. **(D)** Hematoxylin and eosin histological evaluation of the tumor sections from mice treated with 2-DG. Scale bar, 100 µm. **(E)** Tumor-associated macrophages (TAMs) were isolated from tumor tissue by fluorescence-activated cell sorting analysis. The Arg, Fizz, CD206, and Vegf in TAMs were determined by real-time PCR. Data were shown as mean ± SD (*N* = 8). *p < 0.05 and **p < 0.01 compared with the indicated group.

Tumor-associated macrophages (TAMs) are macrophages residing in the tumor microenvironments (3). TAMs (defined as CD45^+^CD11b^+^CD64^+^F4/80^+^ cells) are recognized as M2-polarized macrophages abundantly expressing M2-specific markers such as Arg1, CD206 and high levels of vascular endothelial growth factor (Vegf) (31, 45). To determine aerobic glycolysis on TAMs, we subcutaneously injected 1 × 10^6^ B16 cells, intraperitoneally injected 2-DG from day 9 and harvested on day 19. In the tumor-bearing mouse model, 2-DG significantly inhibited the tumor growth as evidenced by the smaller tumor size and the less tumor weight compared with control tumors (*p* < 0.05, Figures 6A–C). Histological analysis of tumor sections revealed larger necrotic area of tumor in control mice than those in 2-DG treated mice (Figure 6D). Moreover, the sorted TAMs by flow cytometry showed severely reduction of Arg, Fizz, CD206 and Vegf expressions after 2-DG treatment (*p* < 0.05, Figure 6E). In addition, we assessed the direct effect of 2-DG on cell growth of B16 tumor cells *in vitro*. As expected, high doses of 2-DG treatment significantly inhibited B16 tumor cell growth *in vitro*, but low doses of 2-DG (1 mM) failed to do so (Figure S5 in Supplementary Material), indicating that 2-DG can directly inhibit tumor cell growth. These data collectively suggest that 2-DG treatment impacts the polarization of M2-like TAMs in tumor mass *in vivo* while 2-DG has the ability to direct inhibit tumor cell proliferation.

### 2-DG Reduces the Pathogenesis of Allergy Airway Inflammation

**Figure 7:**
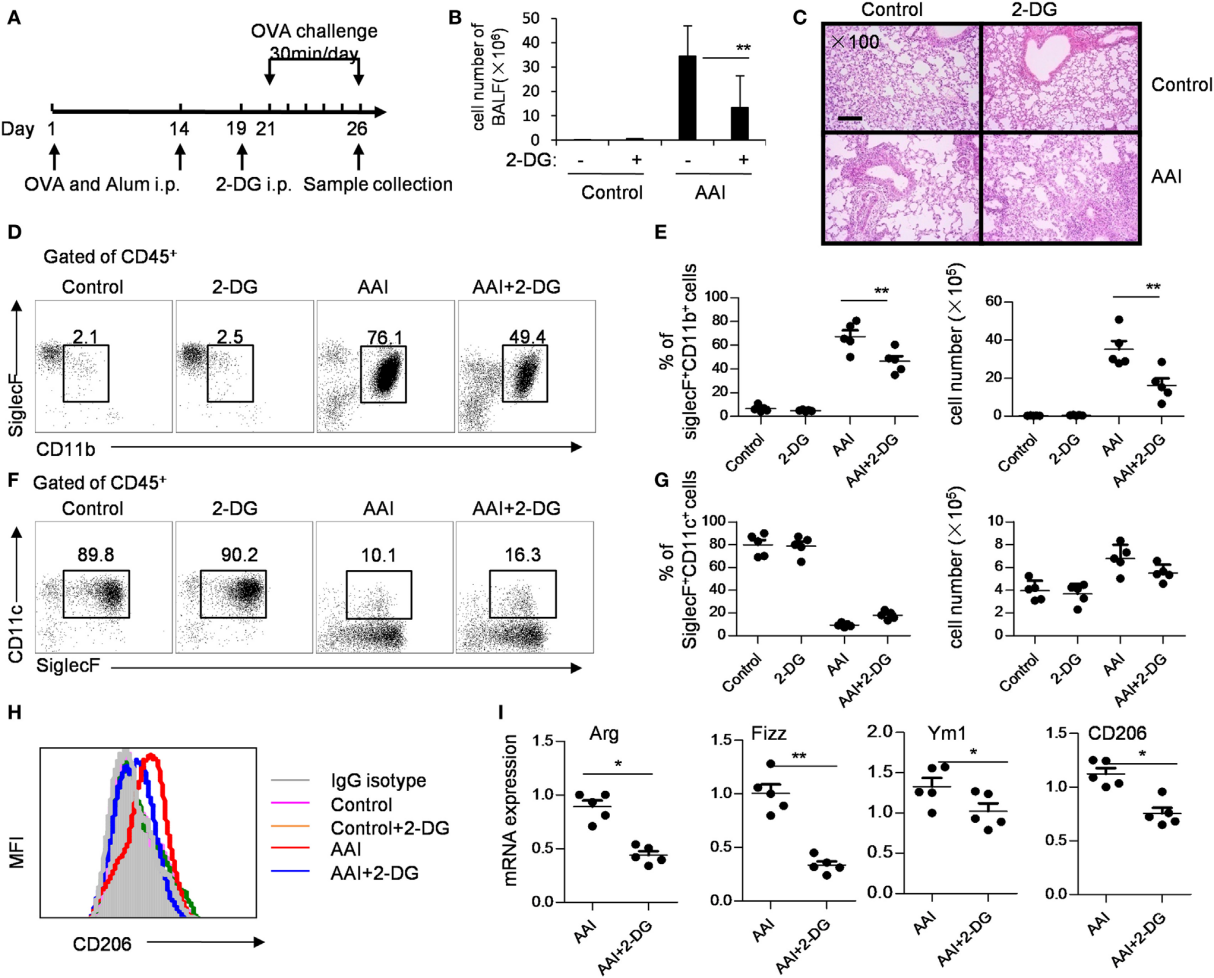
2-Deoxy-d-glucose (2-DG) treatment prevented OVA-induced asthma and M2 polarization. **(A)** OVA-presensitized mice were challenged with aerosolized OVA daily for 5 days as described in materials and methods. The assays were performed on day 6 after OVA challenge. **(B)** Bronchoalveolar lavage fluid (BALF) cell numbers in control and 2-DG treated mice after OVA challenge. **(C)** Hematoxylin and eosin staining of lung tissues of OVA-challenged control or 2-DG-treated mice were presented. Scale bar, 100 µm. Enlarged photos are shown in Figure S6 in Supplementary Material. The flow cytometry staining **(D)** and the percentages and cell numbers **(E)** of infiltrated CD45^+^CD11b^+^SiglecF^+^ eosinophils in BALF of control and 2-DG-treated mice after OVA challenge were shown. The flow cytometry staining **(F)** and the percentages and cell numbers **(G)** of and CD45^+^CD11c^+^SiglecF^+^ macrophages in BALF of OVA-challenged control or 2-DG treated mice were presented. The gate used for different subsets of immune cells was shown in Figure S7 in Supplementary Material. **(H)** The expression of CD206 on CD45^+^CD11c^+^Siglec F^+^ macrophages isolated from BALF of OVA-challenged control and 2-DG-treated mice were analyzed by multicolor flow cytometry. The gate used for CD45^+^CD11c^+^Siglec F^+^ cells was shown in Figure S8 in Supplementary Material. **(I)** The mRNA expressions of Fizz1, Arg1, Ym1, and CD206 in sorted CD45^+^CD11c^+^Siglec F^+^ macrophages isolated from BALF of control and 2-DG-treated mice were detected by real-time PCR. The purity of the sorted CD45^+^CD11c^+^Siglec F^+^ cells was more than 98% (shown in Figure S9 in Supplementary Material). Each assay was performed more than two times. Data are shown as mean ± SD (*N* = 5). *p < 0.05 and **p < 0.01 compared with the indicated group.

In an OVA-induced allergic airway inflammatory mouse model, in which M2 macrophages play a critical role. To address whether 2-DG acts on allergic airway inflammation, we treated OVA-sensitized mice with 2-DG 2 days before the first OVA challenge for 5 consecutive days (Figure 7A). 2-DG injection decreased the cell number in BALF (Figure 7B) and decreased the pathogenesis in lungs as evidenced by H&E staining (Figure 7C; Figure S6 in Supplementary Material). The percentages and cell numbers of eosinophils in the BALF were significantly lower in 2-DG-treated asthma mice than untreated asthma mice (*p* < 0.05, Figures 7D,E; Figure S7 in Supplementary Material), whereas identical cell numbers of the infiltrated macrophages in both mice were observed (Figures 7F,G). Consistent with the previous reports (22), alveolar macrophages of OVA-challenged mice expressed significantly higher CD206 molecules than those of OVA-unchallenged control mice (Figure 7H; Figure S8 in Supplementary Material). However, macrophages from 2-DG-treated allergic airway inflammatory mice showed lower CD206 expression than only OVA-challenged mice (Figure 7H). Moreover, the freshly isolated alveolar macrophages sorted from BALF cells from 2-DG-treated allergic airway inflammatory mice expressed significantly less Arg, Fizz, and CD206 molecules as detected by quantitative PCR (*p* < 0.05, Figure 7I; Figure S9 in Supplementary Material). Thus, 2-DG treatment significantly blocks M2 macrophage polarization and prevent the pathogenesis of allergic airway inflammation in mice.

## Discussion

**Figure 8:**
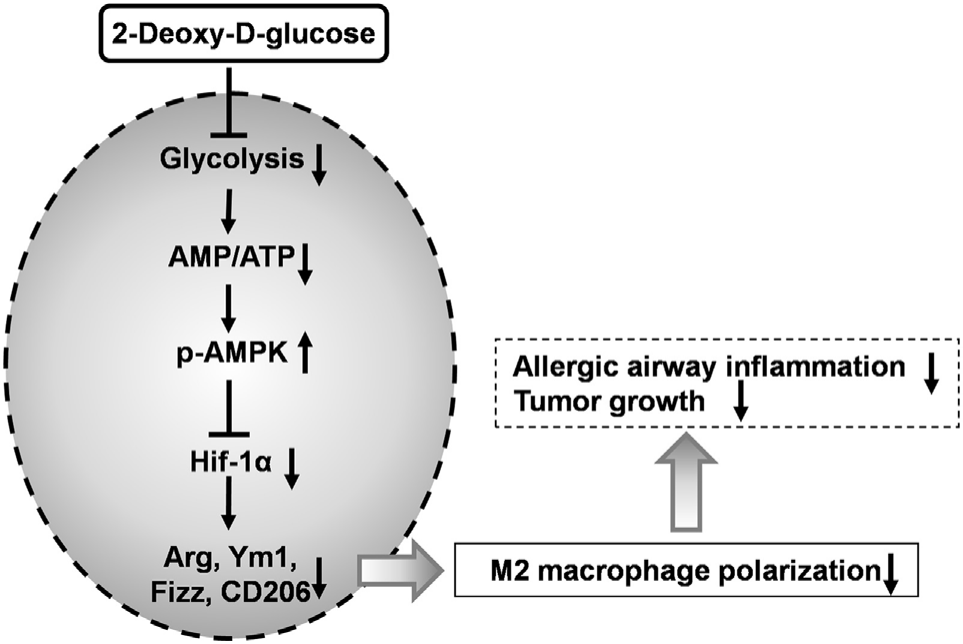
2-Deoxy-d-glucose (2-DG) treatment prevents M2-mediated pathogenesis through an AMPK-Hif-1α-dependent pathway. 2-DG treatment decreased glycolysis in macrophages which then activates AMPK pathway. The activated p-AMPK subsequently inhibits Hif-1α and finally decreased M2 polarized macrophages. 2-DG treatment significantly decreases anti-inflammatory M2 macrophage polarization and prevents disease progression in tumor and allergic airway inflammation.

Macrophages are crucial for immunity and can adopt different activation states. We found that 2-DG treatment prevented not only the glycolysis but also the acquisition of an M2 phenotype *in vitro*. Inhibition of M2 polarization by 2-DG was not due to the cell death. Molecular mechanism studies indicated that the decreased M2 polarized macrophages by 2-DG relies on the activation of AMPK-Hif-1α-dependent fashion (Figure 8). Importantly, 2-DG treatment significantly decreased anti-inflammatory M2 macrophage polarization and prevented disease progression in a series of mouse models with chitin administration, tumor, and allergic airway inflammation. Thus, 2-DG therapy may have beneficial effects in patients with tumors or allergic airway inflammation by its negative regulation on M2 macrophage polarization.

It has been known that while M1 macrophages exert their functions over short time periods, M2 macrophages are engaged in activities that are more prolonged, and FAO is well suited for M2 macrophages to meet the metabolic requirements (7). Although there has been ample evidence showing that FAO is essential in M2 macrophages, there is unclear whether glucose metabolism is involved in this process (46, 47). We found that M2 macrophages had an enhanced glycolysis level as evidenced by the enhanced glucose consumption and lactate production as well as the markedly increased expressions of glycolysis-related genes in IL-4-stimulated M2 macrophages. M2 activation can be blocked by 2-DG, a prototypical inhibitor of the glycolytic pathway via blocking HK. Therefore, glycolysis is necessary for M2 macrophage polarization.

2-Deoxy-d-glucose is a glucose analog and has long been used as a competitive inhibitor of glucose metabolism. 2-DG is taken up through the glucose transporters and then phosphorylated by HK to form 2-DG-6-phosphate (2-DG-6-P). Although glucose-6-phosphate (G-6-P) progresses through the glycolytic pathway, 2-DG-6-P accumulates within the cell and cannot be metabolized further (48, 49). Thus, 2-DG treatment leads to a buildup of 2-DG-6-P in cells to concentrations capable of HK inhibition. 2-DG inhibits mitochondrial ATP production and activates AMPK indirectly by changing cellular AMP: ATP ratio (13). AMPK is a key mediator of a metabolic cell-cycle checkpoint activated by nutrient limitation in mammalian cells (50, 51). Once switched on, AMPK restores energy homeostasis by activating catabolic pathways, while switching off energy-consuming processes such as cell-cycle progression and biosynthesis (50, 52). AMPK activation opposes most of the key metabolic alterations that occur in rapidly proliferative cells and is activated by many existing drugs, including 2-DG by increasing cellular AMP and ADP (13). Hif-1α is a key mediator of AMPK-dependent effects on cellular metabolism (53). The binding of Hif-1α to hypoxia response element-containing promoters were shown to regulate the function of macrophages in response to hypoxia, including the shift to anaerobic enzyme (54–56). Consistent with previous results, AMPK activator stimulation decreased of Hif-1α expression during M2 induction, indicating that AMPK activation inhibited Hif-1α expression. Considering the enhanced AMPK phosphorylation under 2-DG treatment, we concluded that 2-DG decreases M2 polarization via AMPK-Hif-1α pathway.

Usually, solid tumor growth is metabolically active and highly dependent on blood vessels to supply nutrients and to remove metabolic waste. Cancer cells need to acquire diverse metabolic adaptions and stimulate neovascularization to survive and thrive in harsh environments. Studies by Dr. Chuang’s group showed that 2-DG significantly suppressed proliferation, caused apoptosis, and reduced migration of murine endothelial cells (57). Meanwhile, breast cancer stem cells relied on fermentative glycolysis and were sensitive to 2-DG treatment (58). In addition, our present study also showed that high doses of 2-DG significantly inhibited B16 cell proliferation during the *in vitro* culture. Thus, the potential direct effects of 2-DG on tumor growth in tumor-bearing mice in the present study is not excluded. However, manipulation of metabolic reprogramming in immune cells may have therapeutic potential. TAMs usually display an M2-like macrophage phenotype to promote tumor growth directly and via angiogenesis, tissue remodeling, and inhibition of adaptive immunity (59). Tumor-infiltrating T cells such as Th2 cells, immune complexes, or stromal element-derived cytokines like IL-10, and TGF-β can drive TAMs into an M2-like phenotype (60, 61). We found that 2-DG treatment significantly inhibited M2 macrophages polarization in tumors, as evidenced by the decreased Arg, Fizz1, CD206, and Vegf expressions. These results collectively indicate that 2-DG treatment may be beneficial to block the tumor growth through downregulation of M2 macrophage polarization, in addition to its direct inhibitory role on tumor cell proliferation.

Th2 cells and IL-4-driven M2 polarization play active roles in the pathogenesis of allergic asthma (62). 2-DG treatment caused mice resistant to OVA challenge-induced allergic airway inflammation. As compared with allergic airway inflammation control, 2-DG treated mice showed fewer infiltrated immune cells, decreased M2 response including reduced Arg, Ym1, Fizz, and CD206 expressions in macrophages and less infiltrated eosinophils in lungs. Therefore, blocking glycolysis by 2-DG may be used to improve the pathogenesis caused by M2 macrophage differentiation in allergic airway inflammation.

In summary, we demonstrate that glycolysis is essential in M2 macrophage activation and mainly mediated by AMPK-Hif-1α signaling pathway. 2-DG treatment results in impaired M2 macrophage polarization *in vivo* and *in vitro*. The identification of the key role of glycolysis in M2 polarization provides potential molecular targets for the treatment of M2 macrophages-related diseases. 2-DG may be potentially used as a therapeutic strategy to treat cancers and allergy airway inflammation.

## Author Contributions

QZ, ZC, and LZ designed and did the major experiments, analyzed the experimental data, and contributed to the writing. TY designed and analyzed histology and Western blot data. PW designed and performed macrophage isolation and cell culture. FL and YH performed flow cytometry and real-time PCR assays. FZ provided animal models and revised the manuscript. XZ, WD, and YZ designed the experiments, analyzed experimental data, wrote and revised the manuscript, and provided overall direction.

## Conflict of Interest Statement

The authors declare that the research was conducted in the absence of any commercial or financial relationships that could be construed as a potential conflict of interest.
