# Supplementary material for: 2-Deoxy-d-Glucose Treatment Decreases Anti-inflammatory M2 Macrophage Polarization in Mice with Tumor and Allergic Airway Inflammation
Source: Front Immunol. 2017 Jun 1;8:637. doi: 10.3389/fimmu.2017.00637 (PMC5451502; doi:10.3389/fimmu.2017.00637)
Supplement: Supplementary file 1 [file Image_1.PDF]

2-deoxy-D-glucose treatment decreases anti-inflammatory M2 macrophage polarization in mice with tumor and allergic airway inflammation

Qingjie Zhao[1,2,*], Zhulang Chu[1,3,*], Linnan Zhu[1,3,*], Tao Yang[1], Peng Wang[1], Fang Liu[2], Ying Huang[2], Fang Zhang[2], Xiaodong Zhang[4,*, †], Wenjun Ding[2,†], Yong Zhao[1,3,†]

[1] State Key Laboratory of Membrane Biology, Institute of Zoology, Chinese Academy of Sciences, Beijing, China. [2] Laboratory of Environment and Health, College of Life Sciences, University of Chinese Academy of Sciences. Beijing, China. [3] College of Life Sciences, University of Chinese Academy of Sciences. Beijing, China. [4] Department of Urology, Beijing Chaoyang Hospital, Capital Medical University, 8 Gong Ti Nan Road, Chaoyang District, Beijing, China.

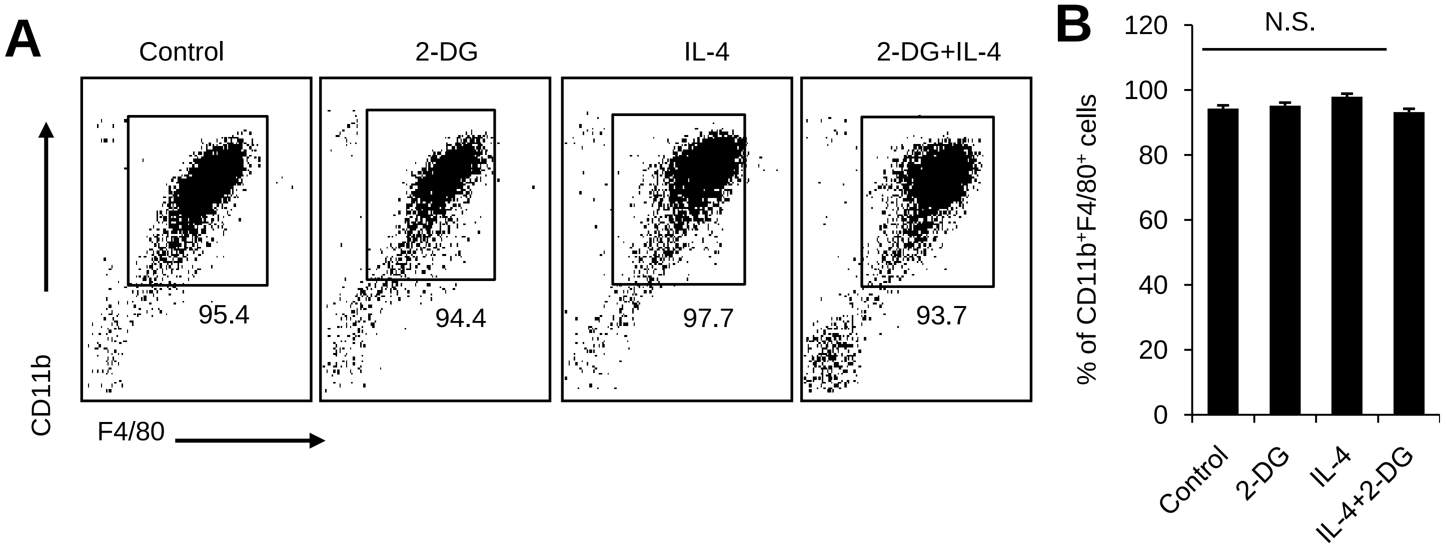

Supplementary Figure 1. Flow cytometry analysis of F4/80 and CD11b expression under the treatment with 2-DG and IL-4 for 48 h.

The freshly isolated peritoneal macrophages were pretreated with 2-DG for 1 h and stimulated with IL-4 for 48 h. The expression of F4/80 and were assayed by flow cytometry (**A**) and percentages of F4/80+CD11b+ cells were summarized (**B**). Data were shown as mean ± S.D. (N = 4). No significance was detected.

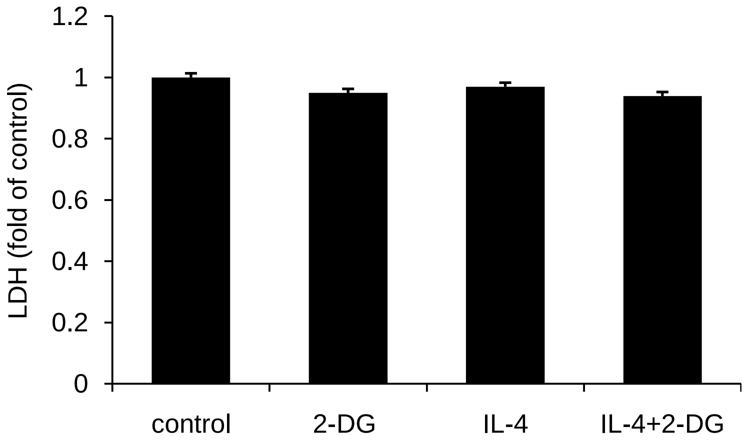

Supplementary Figure 2. LDH release by macrophages cultured in vitro.
The freshly isolated peritoneal macrophages were pretreated with 2-DG for 1 h and stimulated with IL-4 for 48 h. Experiments were done more than two times. Data were shown as mean $\pm$ S.D. (N = 4). No significance was detected.

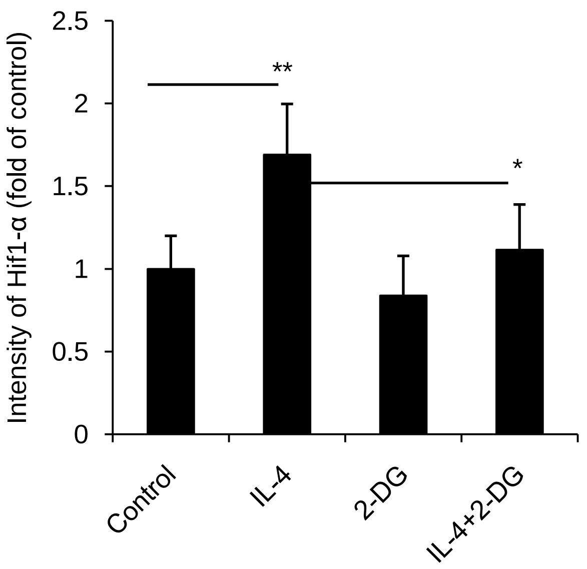

Supplementary Figure 3. Hif1-α protein expression in macrophages treated with IL-4 and/or 2-DG.

The freshly isolated peritoneal macrophages were pretreated with 2-DG for 1 h and stimulated with IL-4 for 48 h. Cell lysates were used to perform Western blot experiments and evaluate protein expression. The intensities of Hif1-α bands in three experiments were summarized. *$p<0.05$, **$p<0.01$ compared with the indicated group.

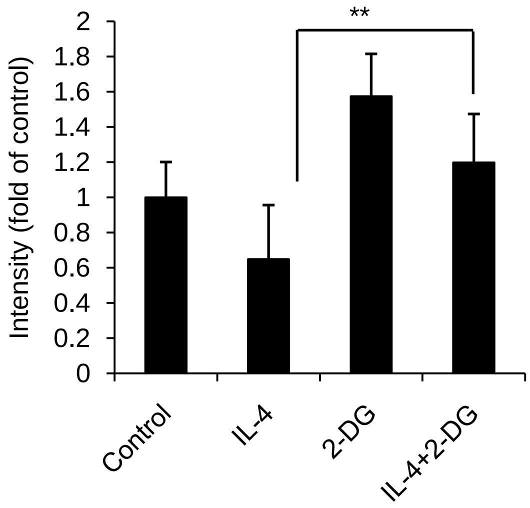

Supplementary Figure 4. The phosphorylated-AMPK protein expression in macrophages treated with IL-4 and/or 2-DG.

The freshly isolated peritoneal macrophages were pretreated with 2-DG for 1 h and stimulated with IL-4 for 48 h. Cell lysates were used to perform Western blot experiments and evaluate protein expression. The intensities of phosphorylated-AMPK bands in three experiments were summarized. **$p<0.01$ compared with the indicated group.

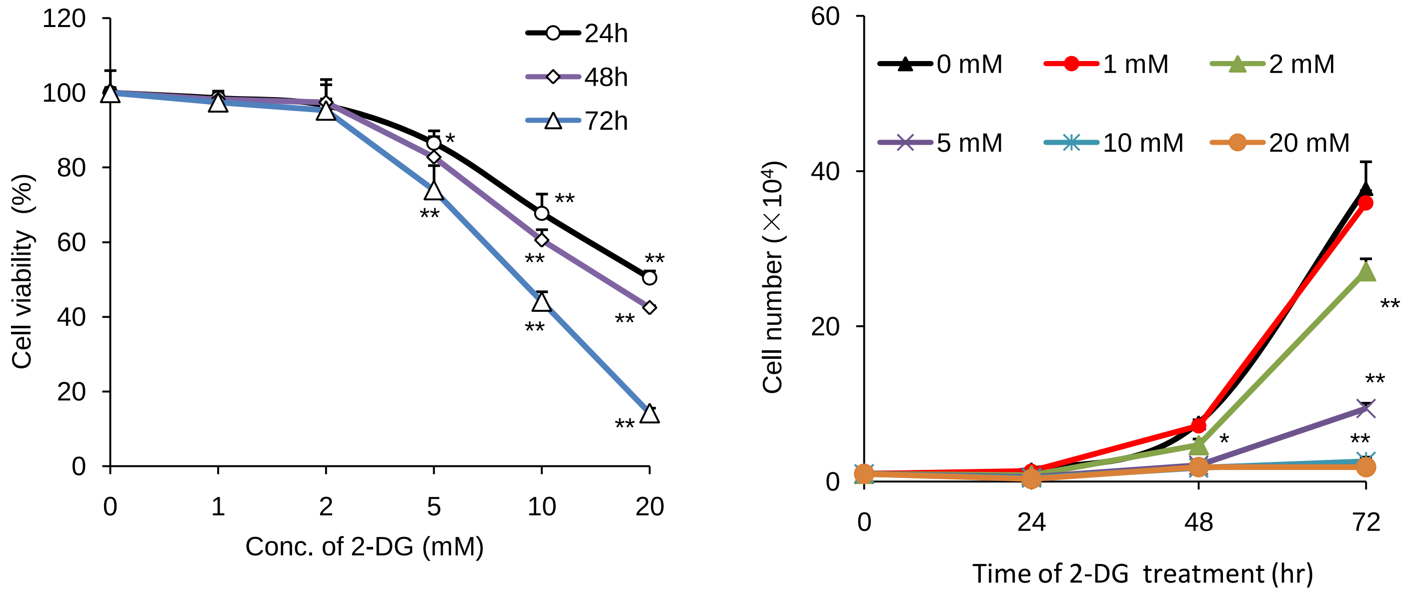

Supplementary Figure 5. The effects of 2-DG on B16 cell survival and proliferation.
The B16 tumor cells were treated with different concentrations of 2-DG (1 mM) *in vitro* for 24 h, 48 h and 72 h. The MTT assay and cell number counts were done. *p<0.05, **p<0.01 compared with the control group which was treated with 0 mM 2-DG.

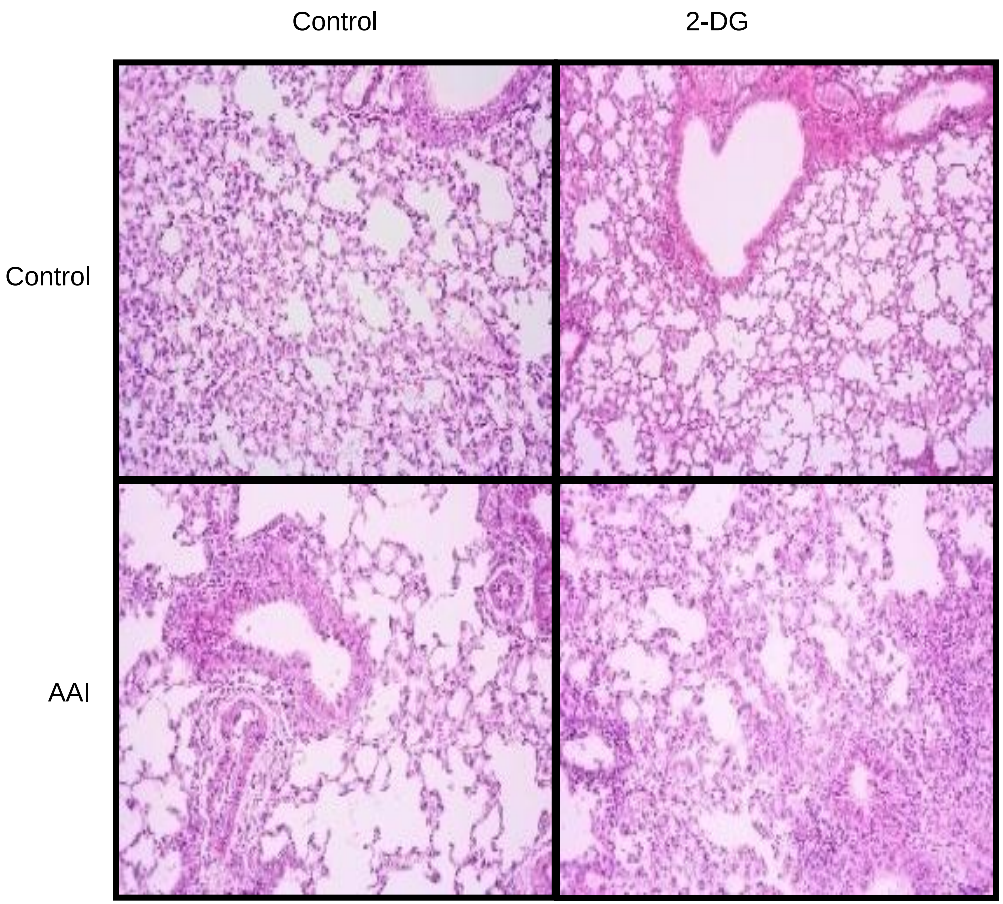

Supplementary Figure 6. H&E staining of lung tissues of control or 2-DG-treated OVA-challenged mice were presented.

More infiltrated of the tracheal and bronchiolar epithelia by numerous inflammatory cells, was observed in OVA-challenged mice compared with 2-DG treated group. OVA-sensitized and challenged mice displayed most bronchi or vessels were surrounded by a thick layer which was improved under the 2-DG treatment.

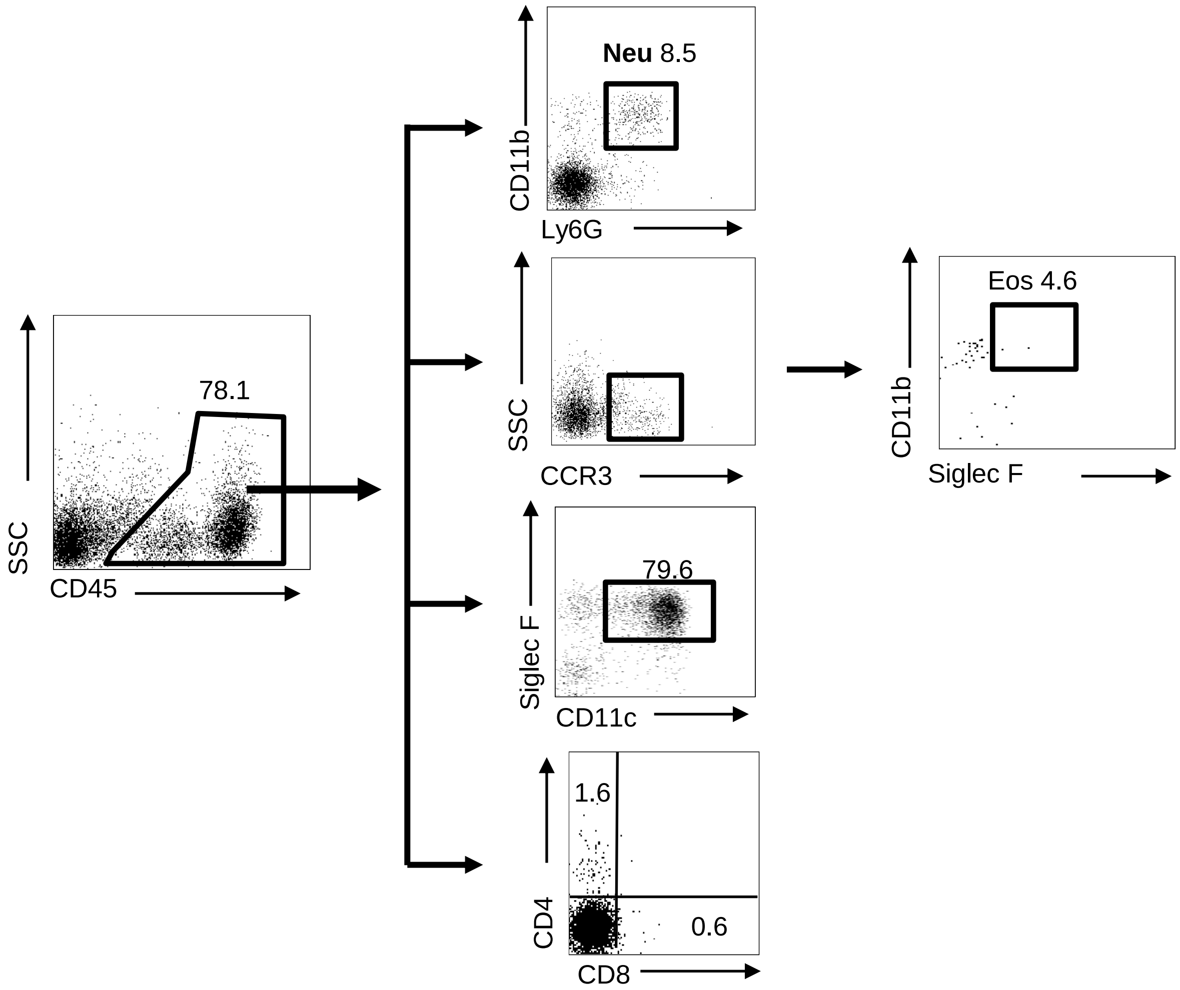

Supplementary Figure 7. To identify immune cell populations in BALF of normal mice by multiple-colors flow cytometry and sequential gating analysis.

After the exclusion of doublets and debris, immune cells were identified using the pan-hematopoietic marker CD45. In normal mouse lungs, a sequential gating strategy was used to identify populations expressing specific markers: alveolar macrophages (SiglecF^{+}CD11c^{+}), neutrophils (CD11b^{+}Ly6G^{+}), eosinophils (SigecF^{+}CCR3^{+}CD11b^{+}), and lymphocytes (CD4^{+}/CD8^{+}).

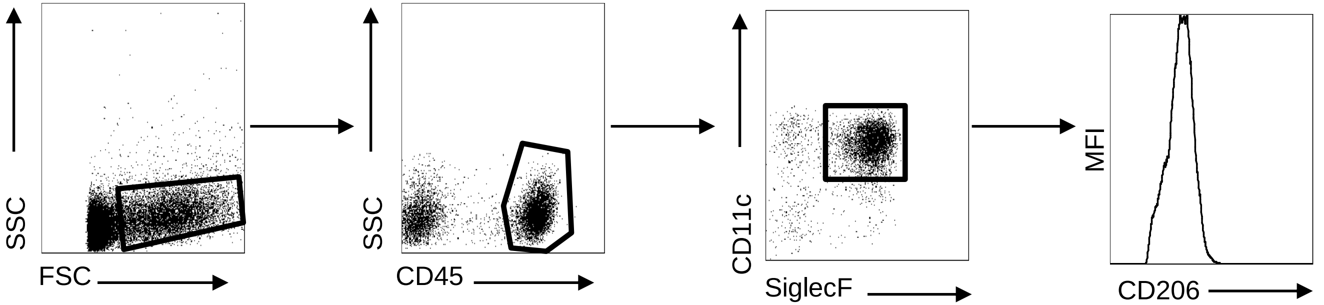

Supplementary Figure 8. Gating for analysis of CD206 expression on CD45+CD11c+SiglecF+ macrophages of BALF.

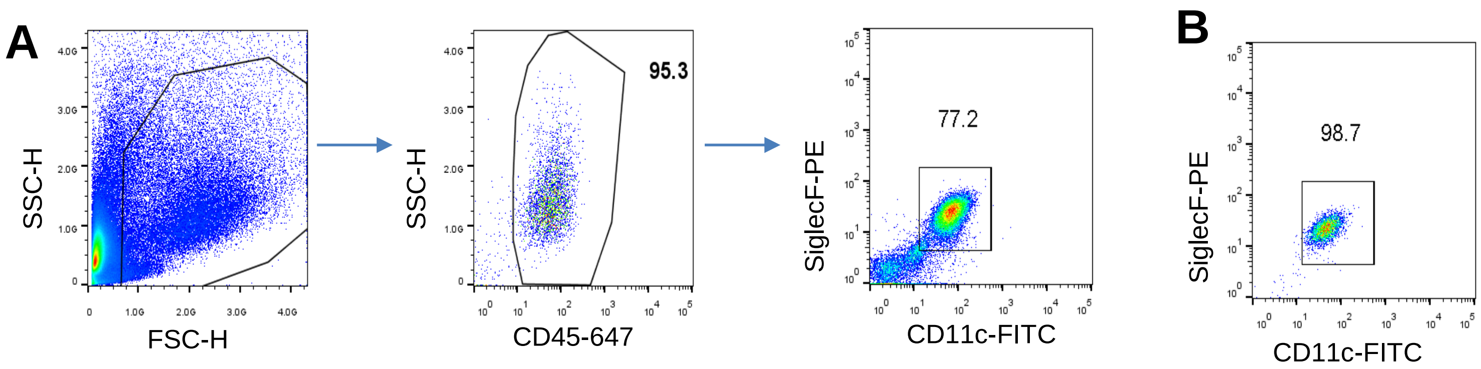

Supplementary Figure 9. Sorting the alveolar macrophages and the cell purity after sorting.
The alveolar macrophages were sorted by flow cytometry (**A**) and the purity of the CD45+CD11c+SiglecF+ macrophages was nearly up to 98% (**B**).
